# Origin, Development, and Synaptogenesis of Cortical Interneurons

**DOI:** 10.3389/fnins.2022.929469

**Published:** 2022-06-27

**Authors:** Alfredo Llorca, Ruben Deogracias

**Affiliations:** ^1^Visual Neuroscience Laboratory, Centre for Discovery Brain Sciences, School of Biomedical Sciences, University of Edinburgh, Edinburg, United Kingdom; ^2^Neuronal Circuits Formation and Brain Disorders Laboratory, Institute of Neurosciences of Castilla y León (INCyL), University of Salamanca, Salamanca, Spain; ^3^Institute of Biomedical Research of Salamanca, Salamanca, Spain; ^4^Department of Cell Biology and Pathology, School of Medicine, University of Salamanca, Salamanca, Spain

**Keywords:** brain, cortex, interneuron, development, synaptogenesis, neuron, cell death

## Abstract

The mammalian cerebral cortex represents one of the most recent and astonishing inventions of nature, responsible of a large diversity of functions that range from sensory processing to high-order cognitive abilities, such as logical reasoning or language. Decades of dedicated study have contributed to our current understanding of this structure, both at structural and functional levels. A key feature of the neocortex is its outstanding richness in cell diversity, composed by multiple types of long-range projecting neurons and locally connecting interneurons. In this review, we will describe the great diversity of interneurons that constitute local neocortical circuits and summarize the mechanisms underlying their development and their assembly into functional networks.

## Introduction

Modern microscopy techniques, together with electrophysiology, and single cell transcriptomics have left behind the times of pen and ink when Santiago Ramón y Cajal, born 170 years ago in Spain, postulated his neuron theory against the established Gerlach's reticular theory.

Today, we know that the brain circuitry consists of mostly two classes of neurons, excitatory projection neurons, and inhibitory GABAergic interneurons (Marín, 2012; Tasic et al., 2016; Tremblay et al., 2016; Lim et al., 2018a). The antagonistic roles of these groups of neurons must be coordinated for the correct function of the circuits in which they are integrated. While cortical projection neurons originate in the pallium, GABAergic interneurons are born from embryonic progenitor cells located in different regions of the subpallium (Xu, 2004; Butt et al., 2005; Fogarty et al., 2007; Miyoshi et al., 2007, 2010; Lee et al., 2010; Rubin et al., 2010; Vucurovic et al., 2010; Gelman et al., 2011). To reach the cortex, these neurons must migrate tangentially following chemoattractive and chemorepulsive external cues (Marín, 2013). After spreading to populate the different cortical areas they switch to radial migration to position into specific cortical layers. Once there, interneurons start to acquire their final morphology, integrate into the brain circuitry, and form their synapses onto the different compartments of surrounding excitatory neurons.

In the present review we will summarize the different mechanisms accounting for the generation of interneuron diversity, their migration to the cortex and their final allocation inside the cortical territory. We will also discuss findings that describe how interneurons integrate in the cortical circuitry and the recently discovered molecular mechanisms that control synapse formation onto their targets.

## Interneuron Diversity in the Mammalian Neocortex

Neocortical circuits are composed of two main neuronal elements: excitatory projection neurons (PNs) and inhibitory interneurons (INs) (Marín, 2012; Tasic et al., 2016; Tremblay et al., 2016; Lim et al., 2018a). PNs are majoritarian, accounting for 70–80% of all neocortical neurons (DeFelipe et al., 2013). These cells release glutamate and connect both with local networks and with physically distant targets, thus mediating communication between different brain regions. In contrast, cortical INs, which account for the remaining 30% neurons in cortex, release GABA, and exclusively establish local connections, primarily regulating the activity of local groups of PNs and other interneurons.

**Figure 1:**
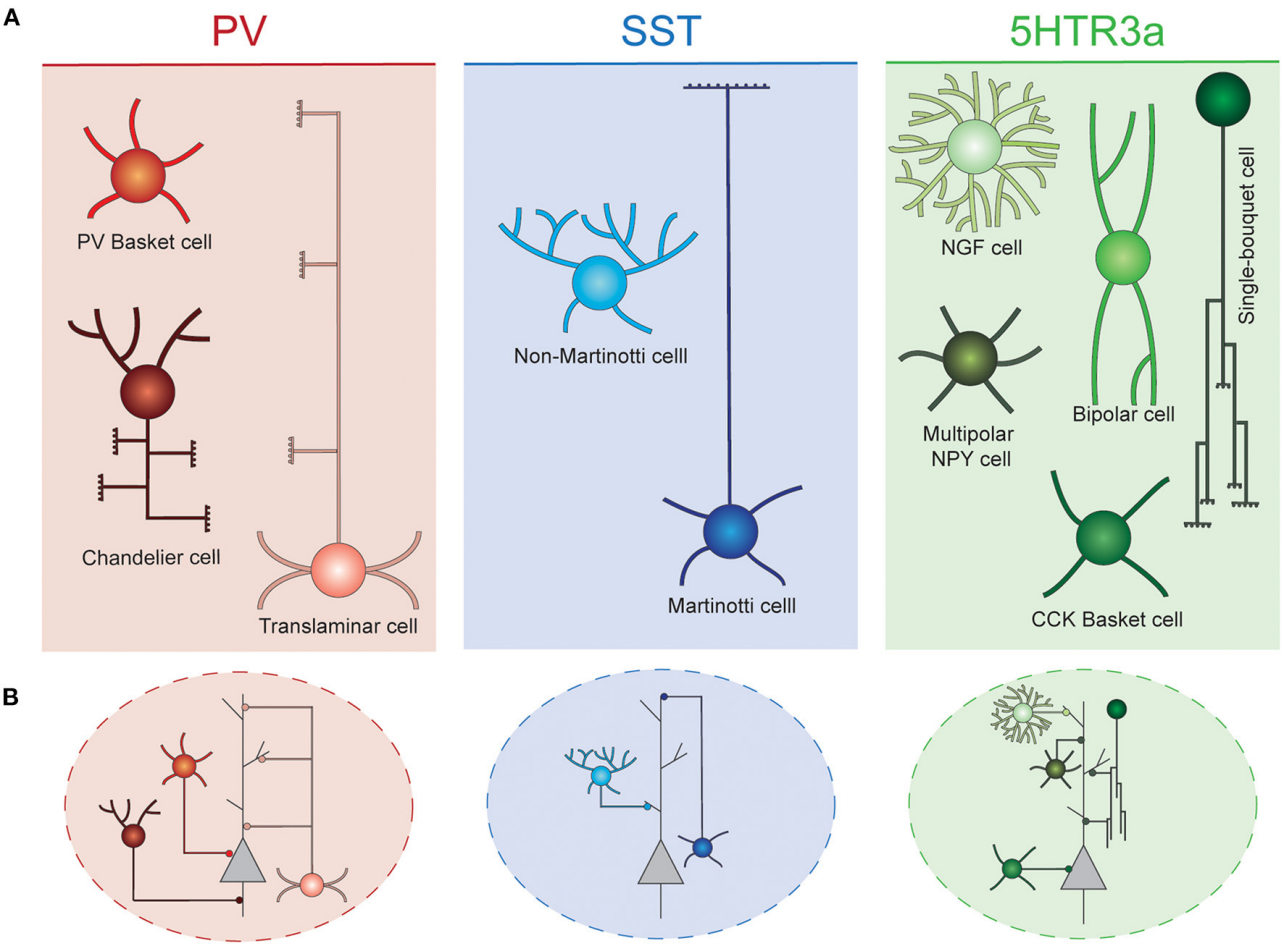
Interneuron diversity in the murine neocortex. **(A)** Cortical interneurons can be classified into three main classes: PV, SST, and 5Htr3a. Each of these classes can be further subdivided into different types. **(B)** Different interneuron types preferentially target specific compartments of surrounding projection neurons. Basket cells target their soma. Chandelier cells specifically innervate axon initial segments. SST and 5Htr3a cells mostly target projection neuron dendrites at different levels. PV, parvalbumin; SST, somatostatin; NGF, neurogliaform; NPY, neuropeptide Y; CCK, cholecystokinin.

Due to their spatially confined connectivity, cortical INs are thought to play key roles in local computations, precisely organizing information flow across groups of surrounding PNs. These cells are extraordinarily diverse, and have been classically classified into different classes based on a combination of morphological, functional and neurochemical criteria (DeFelipe et al., 2013; Tremblay et al., 2016; Lim et al., 2018a). In the rodent cortex, these neurons can be divided into three main groups, known as cardinal classes, that collectively comprise virtually the entire set of cortical INs: Parvalbumin (PV), Somatostatin (SST), and 5HTR3a neurons. Each of these classes can be further subdivided into different interneuron types (Figure 1).

PV cells account for ~40% of all cortical INs, typically target the perisomatic compartment of surrounding PNs and present characteristic fast spiking properties. These neurons can be further subdivided into three main cell types. The most abundant of these are the soma-targeting basket cells. These cells are widely distributed across cortical layers and areas, where their axons target soma and proximal dendrites of surrounding PNs and other INs (Hu et al., 2014). Axo-axonic chandelier cells are a small, yet very important group of neurons. They specifically inhibit the axon initial segment of surrounding PNs, thus strongly controlling the firing activity of those (Somogyi et al., 1982). These cells are most abundant in layer VI and the top border of layer II. Their axonal arbors adopt very characteristic morphologies resembling the shape of a candlestick (Inda et al., 2008). Some chandelier cells do not express parvalbumin (Taniguchi et al., 2013). Finally, translaminar INs represent a third type of PV cells. These neurons are most abundant in deep cortical layers, and extend their axons throughout the entire cortical thickness, targeting PNs of all layers (Bortone et al., 2014). These cells have been proposed to act as a general gain modulation of the entire cortical column (Bortone et al., 2014).

A second major class of cortical INs express the neuropeptide somatostatin (SST). These cells represent ~30% of all cortical inhibitory cells. Two main types of SST neurons populate the cerebral cortex. Martinotti cells constitute a large (~60% of all SST INs) and specialized group of neurons. These cells extend their axons toward neocortical layer I, where they arborize profusely, and inhibit the distal dendrites of PNs (Wang et al., 2004; Hilscher et al., 2017; Maximiliano José et al., 2018). In addition, Martinotti cells also target other INs, mostly PV basket and VIP bipolar cells (Pfeffer et al., 2013). These neurons are most abundant on layer V and II/III, and often express the calcium binding protein calretinin (Xu et al., 2006). They are embedded in a very defined wiring diagram, mostly establishing reciprocal connections with PNs of layers II/III and V (Naka et al., 2019). Martinotti cells can be further subdivided in two subtypes based on their axonal morphology: T-shape and fanning out Martinotti cells (Maximiliano José et al., 2018). Non-Martinotti SST INs lack axonal projections in layer I. Instead, they extend their axons locally, mostly innervating the layer that contain their soma, as well as adjacent layers. They are present across all cortical layers, but are particularly abundant in layer IV, thus showing an inverse laminar distribution to that of Martinotti cells. These cells, establish reciprocal connections with layer 4 granule cells (Naka et al., 2019), and also innervate layer IV basket cells (Xu et al., 2013). Non-Martinotti INs exhibit higher firing frequencies than Martinotti cells but not has high as those characteristic for PV INs (Maximiliano José et al., 2018).

Finally, the third major class of INs, identified by the expression of the serotonin receptor 5Htr3a, accounts for the remaining ~30% of inhibitory cells. This class mostly populates superficial layers of the neocortex and constitutes a diverse conglomerate of cell types with different laminar distribution, morphologies, functional properties, and connectivity patterns. Among these cells, vasointestinal peptide (VIP) expressing bipolar cells are the most abundant. These cells preferentially populate layer II/III and are known to mainly inhibit other INs, thus mediating disinhibition of PNs (Pfeffer et al., 2013; Jiang et al., 2015). They often co-express calretinin and display adapting firing properties (Prönneke et al., 2015). A second type of 5Htr3a neurons also express VIP, as well as cholecystokinin (CCK). These neurons are PV- basket cells and exhibit a multipolar morphology. Their transcriptional profile resembles that of other CCK basket cells that do not express VIP. Remarkably, VIP+ basket cells are most often found in superficial layers, while VIP- basket cells are enriched in deep layers (He et al., 2016). Unlike their PV+ counterparts, these cells exhibit regular or burst firing (Kawaguchi and Kubota, 1998).

Neocortical layer I is most prominently occupied by the distal dendrites of excitatory PNs. Yet it harbors a remarkable diversity of inhibitory cells (Ibrahim et al., 2020). Among these, NDNF+ neurogliaform cells are the most studied. These cells present late spiking properties, and extend tortuous axons that remain confined to layer I, targeting PNs apical dendrites as well as other INs (Letzkus et al., 2011). Previous reports have suggested that, in addition to synaptic transmission, these cells mediate volumetric GABA signaling (Oláh et al., 2007). They receive long range projections from distant brain regions (Letzkus et al., 2011), as well as local inputs from excitatory and other inhibitory cells (Abs et al., 2018). They often express reelin and neuropeptide Y (NPY) (Lee et al., 2010). NDNF+/NPY- canopy cells also confine their axons to layer I (mostly to the upper half of it), although their axonal arborizations are less dense than those of neurogliaform cells (Schuman et al., 2018). Also, they do not share the late spiking properties of their neurogliaform neighbors. Reelin expressing single bouquet cells represent a neuronal population of very characteristic morphology. These cells extend their axons toward deep cortical layers, where they ramify profusely (Jiang et al., 2015). Recent reports have described two distinct subtypes that exhibit this morphology, Alpha-7 and VIP+ layer I interneurons (Schuman et al., 2018). Alpha-7 cells present multipolar dendrites and project to layer V, while VIP cells project to both layers V and VI and present the smallest cell bodies of all L1 neurons. In addition to bipolar and L1 cells, a small population of NPY expressing interneurons populate the top border of layer II (Gelman et al., 2009; Miyoshi et al., 2010). Finally, a small population of Meis2 expressing inhibitory projection neurons reside in the white matter behind the cortex, and innervate deep layers of distant cortical areas (von Engelhardt et al., 2011; Frazer et al., 2017).

In addition to this classic classification of cortical interneuron classes and types, recent efforts have focused in obtaining a complete census of IN diversity in the murine cortex using single cell transcriptomics. These data suggests the existence of around 50 different transcriptional IN types in mouse primary visual cortex (Tasic et al., 2016). Combining transcriptomics with more classic criteria has led to the identification of 28 IN types with discernible morphology, physiological properties and gene expression profiles in this same area (Gouwens et al., 2020). Although the cerebral cortex is composed by diverse regions with remarkable different functions, projection patterns, and cellular architectures, the set of IN types seems to be conserved across areas (Tasic et al., 2018). However, these common cell types have been reported to develop different precise connectivity patterns in different regions of the neocortex, which seem to be acquired *via* divergent developmental trajectories (Pouchelon et al., 2021).

Although this repertory of cell identities was first and most intensely examined in the rodent cortex, recent RNAseq data indicate that it is conserved in primate species, including humans (Hodge et al., 2019). Hence, the core cell classes and types described above can also be found in the primate brain. It is worth noting, however, that the relative abundance of these cell types, their gene expression patterns, morphologies and distribution in different cortical regions and layers present remarkable differences in the primate brain (Hodge et al., 2019; Krienen et al., 2020).

## Genesis of Cortical Interneurons

In contrast to PNs, which are produced in the dorsal pallium, cortical INs are born in the ventral subpallium, far from their adult location. Three main subpallial structures source cortical interneurons: the medial ganglionic eminence (MGE) the caudal ganglionic eminence (CGE) and the preoptic area (POA). Nevertheless, the cellular mechanisms sustaining IN production generally resemble those underlying the genesis of their excitatory counterparts. Remarkably, recent studies using human tissue indicate that a subpopulation of inhibitory INs is derived from cortical radial glial cells (RGCs), the main progenitor cells sourcing excitatory neurons (Delgado et al., 2021), a phenomenon that is not observed in the mouse brain (Bandler et al., 2022).

**Figure 2:**
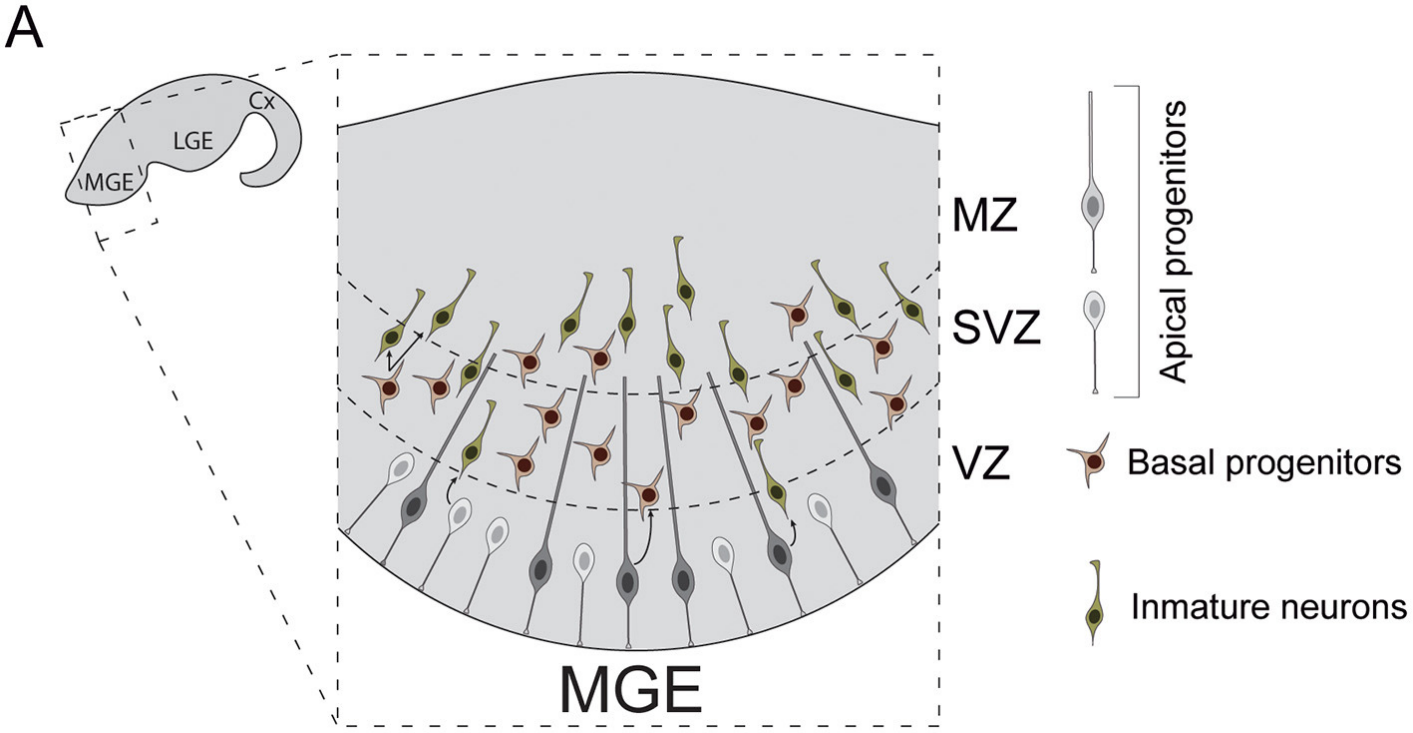
Neurogenesis in the mouse medial ganglionic eminence. **(A)** Diverse types of progenitor cells underlie the genesis of cortical interneurons in the embryonic subpallium. Apical progenitors reside in the VZ and divide to generate neurons and basal progenitors. Basal progenitors migrate to the SVZ where they divide to generate post-mitotic interneurons. These neurons then migrate to the MZ and leave the MGE to invade the developing cortex. Cx, cortex; LGE, lateral ganglionic eminence; MGE, medial ganglionic eminence; VZ, ventricular zone; SVZ, subventricular zone; MZ, marginal zone.

The subpallial neuroepithelia can be subdivided into three main laminae: ventricular zone (VZ), subventricular zone (SVZ) and marginal zone (MZ) (Figure 2). Diverse populations of progenitor cells reside in these regions. Apical progenitors populate the VZ. Some exhibit bipolar morphologies that resemble those of cortical RGCs (Brown et al., 2011). Unlike those, the basal process of subpallial apical progenitors not always reach the pial surface (Tan et al., 2016), often anchoring to blood vessels instead. Others present apical but not (or very short) basal processes, similar to pallial short neural precursors (Petros et al., 2015). Apical progenitors divide at the ventricular surface to generate both basal progenitors and cortical interneurons (Brown et al., 2011; Ciceri et al., 2013). Unlike cortical RGCs, some apical progenitor cells are known to divide inside the VZ, away from ventricular surface (Pilz et al., 2013). These cells have been studied in depth in the lateral ganglionic eminence (LGE), a subpallial structure that does not contribute to the generation of cortical INs. In this structure, these stem cells have been proposed to constitute a separated category, named sub-apical progenitor cells, which are quite diverse in morphology (Pilz et al., 2013). Whether similar populations of sub-apical progenitor cells can be found in the subpallial structures that source cortical INs, such as the medial or the caudal ganglionic eminences, remains to be investigated.

Basal progenitors also participate in the genesis of cortical INs. These cells originate from apical progenitor divisions. Unlike those, basal progenitors acquire multipolar morphologies and migrate toward the SVZ, where they divide to expand interneuron lineages (Ciceri et al., 2013). In the human developing brain, the subpallial SVZ is remarkably expanded and can be divided into inner and outer subventricular zones (iSVZ, oSVZ). This enlarged SVZ also exhibits increases in the number of basal progenitor cells compared to rodents (Hansen et al., 2013). Indeed, as development progresses, human MGE VZ seems to get thinner and SVZ thicker, with basal neurogenesis becoming dominant after gestational week 10 (Hansen et al., 2013). Moreover, nests of proliferating neuroblasts have been recently reported inside human MGE oSVZ (Paredes et al., 2022). These neuroblasts exhibit sustained proliferation, which can account for the production of the larger number of INs that populate the adult human cortex.

## Fate Specification of Cortical Interneurons

Remarkable efforts have been made in the last decades to understand the developmental origins of INs diversity. A key question here is when does a neuron decide its adult fate. It seems clear that INs mature slowly during their first weeks of life, progressively developing their adult features, and only acquiring their adult fate after settling in the cortex. Also, they often need to establish physical and functional interactions with developing neuronal networks to develop properly (Kepecs and Fishell, 2014; De Marco García et al., 2015; Wamsley and Fishell, 2017; Mossner et al., 2019; Wester et al., 2019). Indeed, during early postnatal life, local environment seems to influence the identity of neocortical and hippocampal interneurons (Quattrocolo et al., 2017). Nevertheless, strong evidences also suggest that INs are directed toward the acquisition of their adult fate shortly after birth, at least in respect to their class identity (Bandler et al., 2022). New-born INs in the MGE already present important differences in gene expression patterns (Mayer et al., 2018; Mi et al., 2018), and chromatin opening of distal regulatory elements (Allaway et al., 2021). These differences are linked to differentiation toward a particular class identity. Moreover, both cell culture and transplantation experiments have revealed that new-born INs are capable to differentiate into specific classes despite not been exposed to the natural developmental process (Xu, 2004; Alvarez-Dolado et al., 2006; Wonders et al., 2008; Inan et al., 2012; Howard and Baraban, 2016). Finally, SST+ Martinotti cells seem to exhibit differential migratory behaviors prior acquisition of their adult fate, indicating that these cells are already segregated from other populations before invasion of the cortical territory (Lim et al., 2018b; Mayer and Fishell, 2018). Thus, cortical interneurons seem to be determined to follow a differentiation route shortly after they are born, but progressively unfold their developmental programs as development proceeds (Allaway et al., 2021), interacting with their surroundings to refine their adult properties.

The mechanisms that account for the generation of cortical IN diversity have been extensively studied. As is the case in other regions of the nervous system, such as the spinal cord or the dorsal pallium, a combination of spatial and temporal patterning mechanisms play key roles in this process.

### Spatial Specification of Cortical Interneurons

**Figure 3:**
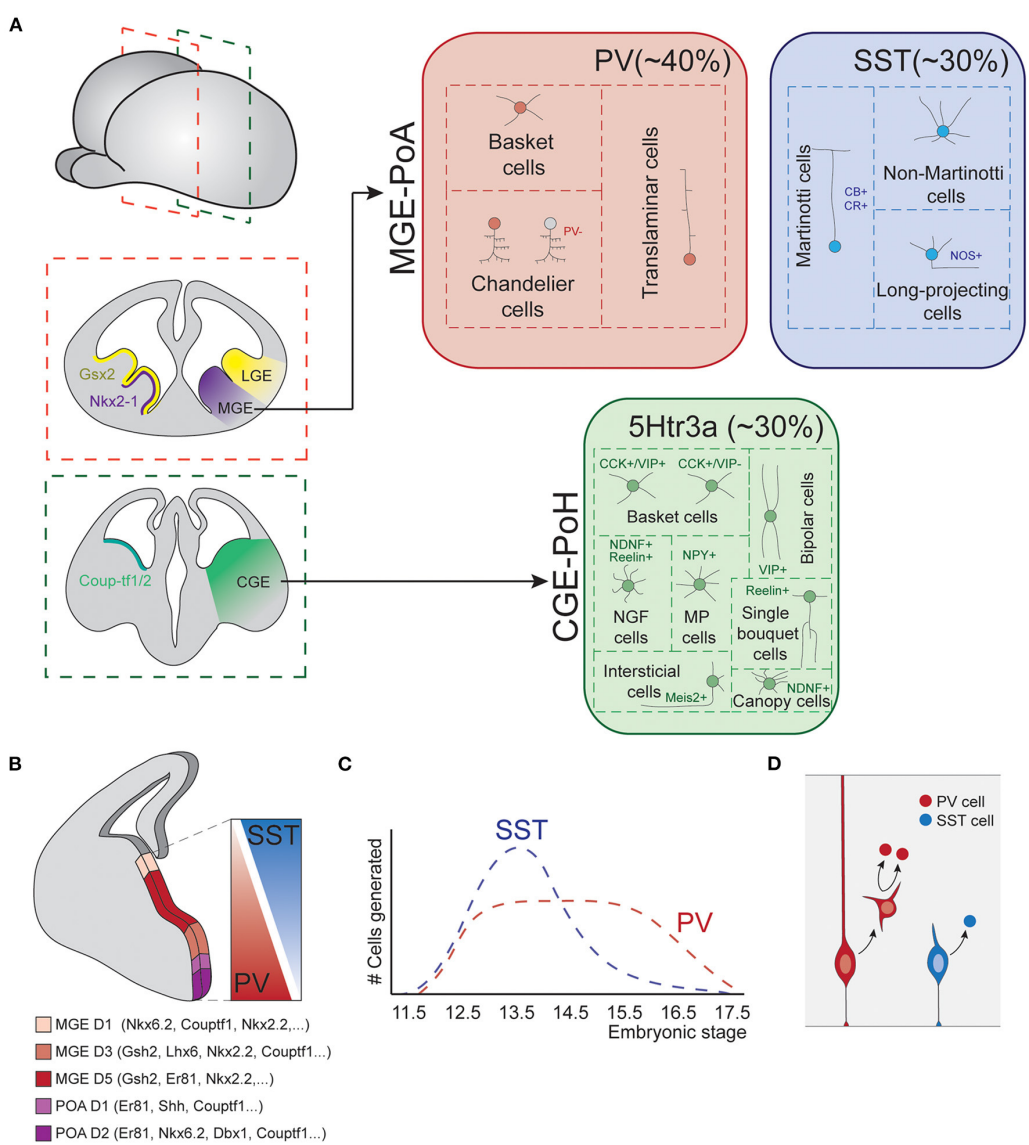
Fate-specification of cortical interneurons. **(A)** The spatially organized expression of specific combinations of transcription factors subdivides the subpallium into different structures. Among those, the MGE and POA generate interneurons of both PV and SST classes. The CGE and POH are responsible for the generation of all types of 5Htr3a neurons. **(B)** MGE is further subdivided into spatial domains in the dorsoventral axis, each of these domains is defined by differential gene expression patterns. Such spatial patterning relates to the production of different interneuron types, as SST and PV cells largely derive from dorsal and ventral MGE respectively. **(C)** Temporal biases in interneuron origin. SST cells are mostly produced during early neurogenesis, while PV cell production remains nearly constant throughout the entire neurogenic window. **(D)** Progenitor cell mode of division also influences interneuron fates. Direct neurogenesis from apical progenitors mostly produces SST cells, while basal progenitor divisions mostly generate PV fates. MGE, medial ganglionic eminence; POA, preoptic area; CGE, caudal ganglionic eminence; POH, Preoptic-hypothalamic border domain; PV, parvalbumin; SST, somatostatin; CB, calbindin; CR, calretinin; NOS, nitric oxide synthase; CCK, cholecystokinin; VIP, vasointestinal peptide; NDNF, neuron derived neurotrophic factor; NPY, neuropeptide Y.

Prior to the start of neurogenesis, the action of diverse morphogen signals divides the ventral subpallium into different territories, each of which is specialized in producing different types of neurons (Figure 3A; Xu, 2004; Butt et al., 2005, 2008; Flames et al., 2007; Gelman and Marín, 2010; Xu et al., 2010; Mayer et al., 2018).

The most dorsal division of the subpallium originates the LGE. This structure is the major source of striatal GABAergic projection neurons but does not produce cortical INs. The most ventral division expresses Nkx2.1 and originates the medial ganglionic eminence (MGE) and the preoptic area (POA). These structures are responsible for the generation of two major IN classes: PV and SST cells (Lavdas et al., 1999; Wonders and Anderson, 2006; Xu et al., 2008; Gelman et al., 2009, 2011; Marín, 2013), as well as long-range projecting nNOS+ neurons (Magno et al., 2017). In addition, the most caudal part of the LGE, acquires very different neurogenic properties. This structure does not present a defined physical boundary with the rest of the LGE but is clearly segregated by differential gene expression patterns. This region is known as the caudal ganglionic eminence (CGE) and originates INs of the third major IN class, the 5HTR3a expressing cells (Nery et al., 2002). Ventral to the CGE, the preoptic-hypothalamic border domain (POH) exhibits gene expression patterns that resemble those of CGE, despite being a caudal extension of the POA. This region mostly produces neurogliaform cells and NPY multipolar INs (Niquille et al., 2018). Although most of our current knowledge about the development of IN diversity arises from studies in the murine brain, recent data indicates that these principles are generally conserved in primates (Hansen et al., 2013; Ma et al., 2013).

Remarkably, each of these structures is still capable to generate diverse interneuron types. This is in part due to further spatial patterning events that subdivide these structures into smaller domains with differential neurogenic capabilities.

In the MGE, several transcription factors show differential expression along the dorso-ventral axis, delineating up to five different gene expression domains (Figure 3B; Flames et al., 2007). Perhaps the best example of such differential expression are the transcription factors *Nkx6.2* and *Er81*, which are expressed in opposite dorso-ventral and ventro-dorsal gradients, respectively. These dorsoventral patterning of the MGE seem to be linked to the production of different IN types. Hence, dorsal Nkx6.2 rich domain primarily produces SST+ neurons, while ventral Er81 rich domain generates a major fraction of PV INs (Fogarty et al., 2007; He et al., 2016). Transplantation experiments of dorsal or ventral patches of donor MGE tissue into host subpallium further support this view. Neurons derived from dorsal donor tissue mostly acquire SST fates while those derived from ventral MGE tissue mostly differentiate into PV cells (Flames et al., 2007; Wonders et al., 2008). Other classic spatial patterning cues, such as Shh, have also been reported to influence IN specification, favoring SST cells production at the expense of PV neurons (Xu et al., 2010).

Although patterning mechanisms in the MGE rostro-caudal axis have been less studied, differences in gene expression along this axis have also been linked to the genesis of different IN classes. The transcription factors Couptf1/2 are expressed in the most caudal aspect of MGE, a spatial domain that generates dominant fractions of SST cells (Hu et al., 2017).

Evidence of spatial patterning inside the CGE has also been reported. A study from the Muramaki group observed striking differences in the IN fates derived from indiscriminately targeting CGE regions using *in utero* electroporation (Torigoe et al., 2016). These observations suggest that different spatial regions of CGE are competent for the genesis of different IN types.

Taken together, these evidences indicate spatial segregation of progenitor cell pools with different neurogenic potential within the major subpallial structures that source cortical INs. However, this segregation does not seem to be sharp nor complete. The different MGE spatial domains, for instance, exhibit clear biases in the production of different IN classes, but still produce multiple cell classes and types. Therefore, additional mechanisms should help to finally delineate specific IN identities. While these could include further spatial segregation of smaller progenitor cell domains yet unidentified, other kind of mechanisms, such as temporal patterning, are known to participate in the specification of cortical IN identity.

### Temporal Specification of Cortical Interneurons

In the dorsal pallium, different types of excitatory projection neurons are generated in strict sequential order. In fact, it has been proposed that radial glial progenitor cells progress through sequential windows of competence as development proceeds and are capable to generate specific neuron types during each of these competence windows. This model of neurogenesis is commonly known as a progressive restriction (Molyneaux et al., 2007; Greig et al., 2013; Oberst et al., 2019; Lin et al., 2021; Llorca and Marín, 2021). Whether subpallial progenitor cells use similar mechanisms to produce IN diversity remains unclear, but clear temporal biases in the production of different IN identities have been reported.

SST INs are generated in larger numbers at early developmental stages, while PV cells are produced at nearly constant rates throughout the neurogenic period (Figure 3C; Miyoshi et al., 2007; Inan et al., 2012). In addition, temporal biases have also been observed in the genesis of precise types. Chandelier cells represent an excellent example of this, since they are specifically produced during late neurogenesis (Taniguchi et al., 2013). Although less understood, neurogenesis in the CGE also seem to be temporally organized, with different ratios of cell types generated in early vs. late neurogenesis (Miyoshi et al., 2010).

Temporal mechanisms have also been linked to the production of IN laminar fates. Mirroring their excitatory counterparts, MGE-derived cortical INs are generated in an inside-out pattern that correlates with birthdate (Valcanis and Tan, 2003; Pla et al., 2006; Rymar and Sadikot, 2007). This is consistent with the mentioned SST-PV ratio gradient, since SST cells are more abundant in deep layers, while PV cells are distributed across all layers. A recent study suggest that this inside-out gradient is only part of a more complex inside-outside-in dynamic where production of deep layer INs is enriched during early and late neurogenesis, and superficial layer neurogenesis is enhanced in between (Sultan et al., 2018). It is worth noting that CGE neurogenesis does not seem to exhibit these dynamics. Indeed, it maintains a constant ratio of enriched production of superficial layer neurons (Miyoshi and Fishell, 2011).

Analysis of single progenitor cell outputs has also contributed to our understanding of IN development. Individual apical progenitor cells in the murine MGE are competent to generate multiple IN types, including cells of both SST and PV classes (Brown et al., 2011; Ciceri et al., 2013; Harwell et al., 2015; Mayer et al., 2015; Bandler et al., 2022). This is consistent with a progressive restriction model of neurogenesis. In contrast, however, the generation of laminar identities seems partially segregated in different progenitor cell pools. Hence, while some single-progenitor derived IN clones expand across layers, most of them seem to be confined into one or two adjacent layers (Ciceri et al., 2013). These findings suggest a mixed model of neurogenesis, in which specialized progenitor pools are committed to generate specific IN laminar fates but can generate both SST and PV class identities. Such multipotency could be achieved through temporal progression of internal genetic programs as described in pallium, although such programs have not yet been described in detail. Intriguingly, these clones where reported to constitute spatial clusters in adult cortex, suggesting that individual progenitor cells in the MGE were also somehow tuned to produce neurons destined to specific cortical regions (Brown et al., 2011; Ciceri et al., 2013). This view was challenged by later studies which used genetic barcoding to identify lineage related neurons occupying distant locations (Harwell et al., 2015; Mayer et al., 2015). The spatial dispersion of MGE derived IN clones thus remains controversial, and further studies will be needed to fully understand these matters.

### Mitotic Patterns and Interneuron Fate Specification

In addition to spatial and temporal patterning mechanisms, other factors contribute to the generation of cortical IN diversity. Petros et al. proposed that mode of progenitor cell division influences the identity of nascent progenies. Hence, apical progenitors which produce neurons through direct neurogenesis mostly generate SST INs, while divisions of basal progenitors will mainly generate PV cells (Figure 3D; Petros et al., 2015). In line with these findings, *Cyclin D2* knockout mutants, which show reduced mitotic activity at MGE SVZ (Glickstein et al., 2009), present a severe loss of PV INs (Glickstein et al., 2007). Also, the transcriptions factors *c-Maf* and *Maf-b*, which are expressed in the SVZ of the embryonic MGE, are known to suppress SST cell identity and promote differentiation into PV fate (Pai et al., 2019). Consistently, knockout mutants of these genes exhibit an increase in SST cell numbers at the expense of PV cells in hippocampus and neocortex.

How this integrates with the spatial and temporal patterning mechanisms described above remains to be clarified. Individual progenitor cells could switch their preferred mode of division as neurogenesis proceeds, with direct neurogenic divisions dominating during early neurogenesis, and indirect neurogenesis taking over at later stages. This would account for the temporal biases observed in the production of those identities, as well as for progenitor multipotency. Also, different spatial domains could contain populations of progenitor cells with different preferences for mode of division.

In sum, diverse mechanisms act in combination to specify the identities of nascent neurons in the embryonic subpallium. While spatial and temporal patterning play dominant roles, other mechanisms also help refine this process. Shortly after birth, INs migrate toward the cortex (Marín and Rubenstein, 2001; Bartolini et al., 2013), leaving subpallium behind and commencing a long journey that will further refine the diverse features that constitute their adult fate.

## Interneuron Migration to the Pallium

Precise developmental programs orchestrate the assembly of both projection neurons and interneurons into functional neocortical circuits [review by Bartolini et al. (2013), Tremblay et al. (2016), Silva et al. (2019)]. Remarkably, PNs originate in the presumptive neocortex, while cortical INs originate in the ventral subpallium, far from this structure.

Thus, once generated in the subpallium, interneurons need to migrate long distances to reach their final position in the pallium. Despite the diversity of INs and their different origin, all the INs share a similar 3-steps migration to reach the cortex with multiple similarities: (1) INs tangentially migrate into the cortex (2) tangentially disperse along all the cortical areas and (3) reach their final location and integrate into specific cortical layers by radial migration.

**Figure 4:**
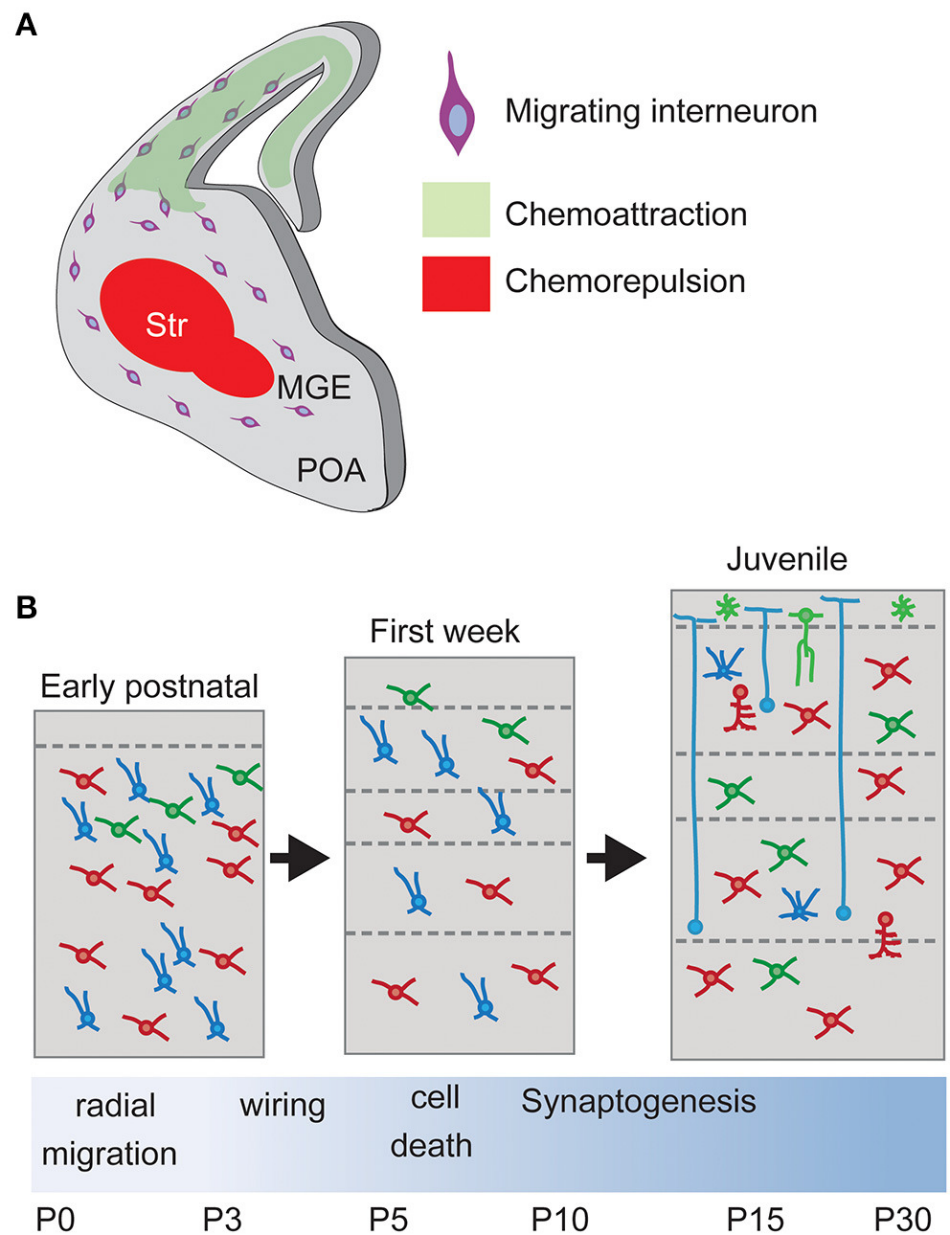
Interneuron migration and integration. **(A)** Upon born, non-mitotic cortical Interneurons migrate through the subpallium avoiding entering the Striatum, a restrictive area due to chemorepulsive cues, and following a chemoattractive gradient of cues until reaching the final positioning in the cortex. Str, striatum. **(B)** Laminar allocation and integration into cortical circuits. During the first week, Interneurons allocate into their final laminal position following a period of programmed cell death. Surviving neurons develop synapses according to their type onto Pyramidal Neurons.

The migration process depends on a complex and coordinated transcriptional program of external guidance molecules and cell surface receptors. External molecular cues are expressed along the different migratory pathways in order to direct the migration of the INs to their final destiny. At the same time, each subtype of IN deploys its own transcriptional program to express specific receptors and internal signaling pathways. In this way, different INs select different migratory tracks in response to the guidance cues. The coordination of these two programs is essential to achieve proper integration and neuronal circuit formation (see Figure 4 for overview).

Newborn INs respond to different cues during their tangential migration to the pallium. These factors include neurotrophins and neurotrophic factors such as Brain Derived Neurotrophic Factor (BDNF), Neurotrophin-4 (NT-4), Glial Cell-derived Neurotrophic Factor (GDNF), Hepatocyte growth factor (HGF) as well as the neurotransmitters GABA, Glutamate and Dopamine.

Tropomyosin-receptor kinase B (TrkB) is the high affinity receptor for both BDNF and NT-4. *In vitro* experiments have shown that the activation of TrkB by both neurotrophins strongly promote IN migration in organotypic slice cultures (Polleux et al., 2002). Inhibition of downstream PI3-kinase but not MAPK- or PLC-gamma pathways dramatically attenuates tangential migration of IN. However, the deletion of TrkB in a mutant mouse model did not show a change in position of INs, despite that it shows a direct regulation of the synaptically enriched GAD65 protein *via* the MAPK/CREB pathway (Sánchez-Huertas and Rico, 2011).

The action of GDNF during IN migration seems to be mediated by the GDNF Family Receptor alpha1 (GFRa1) and the proteoglycan syndecan-3 (Canty et al., 2009; Bespalov et al., 2011). This pathway is independent of the co-receptor signaling molecules Rearranged During Transfection (RET) and Neural Cell Adhesion Molecule (NCAM) (Pozas and Ibáñez, 2005; Canty et al., 2009). *In vivo* data obtained from GFRa1 mutant mice has shown that the role of GDNF could be beyond IN mobility. These mice showed a regionalized loss of PV interneurons, thus suggesting that GFRa1 contributes to the diversification and allocation of specific cortical interneuron subtypes (Canty et al., 2009).

HGF and its receptor MET have also a strong effect on IN migration but only *in vitro*, as MET seems to be expressed only by MGE-derived interneurons under cultured conditions but not *in vivo* (Powell et al., 2001; Eagleson et al., 2011).

GABA and glutamate migratory effect on INs is mediated by the activation of their GABA and glutamate AMPA receptors, respectively, which are highly expressed by INs soon after they start their migration (Cuzon et al., 2006; Manent et al., 2006; Bortone and Polleux, 2009). Contrary to the membrane hyperpolarization caused by GABA in adult neurons, marked by the upregulation of the potassium and chloride exchanger KCC2 after migration, GABA causes membrane depolarization on migrating INs (Bortone and Polleux, 2009). This is due to the activation of L-type voltage-sensitive calcium channels (VSCCs), which stimulates interneuron activity. Inhibition of GABA signaling causes accumulation of INs at the subpallial/pallial border, preventing them from entering the pallium. Glutamate also causes membrane depolarization and calcium transients (Métin et al., 2000; Bortone and Polleux, 2009). Activation of L-type voltage-sensitive calcium channels (VSCCs) also stimulate interneuron motility.

Dopamine, secreted in the LGE by the thalamo-striatal axons during the embryonic period, causes activation of dopamine receptors D1 and D2 expressed by INs. While activation of D1 promotes migration, D2 activation causes IN stop (Ohtani et al., 2003), indicating an opposite effect of these receptors in response to the same external cue.

During their migration from the subpallium and while attracted to the cortex by several diffusible molecules, interneurons avoid transiting through the POA and the striatum, immediately adjacent to the Ganglionic eminences. This is due to the action of chemorepulsive cues secreted by the POA and striatum (Marín et al., 2003; Nóbrega-Pereira et al., 2008; Hernández-Miranda et al., 2011). *In vitro* experiments have shown that diverse molecules mediate this chemorepulsive effect. Striatum and POA express semaphorins Sema3A and Sema3B, which can be sensed by Robo1 receptor expressed by cortical INs. The interaction between ephrinA and their receptors also participates (Marín et al., 2001; Nóbrega-Pereira et al., 2008; Hernández-Miranda et al., 2011).

While cortical INs avoid entering the POA and striatum, they are attracted to the cortex following a gradient of permittivity. The two isoforms of Nrg1 (Neuregulin-1), the membrane-bound protein CRD-Nrg1 and the secreted isoform Ig-Nrg1, act as long-range and short-range chemoattractant cues from the pallium (Flames et al., 2004; Martini et al., 2009). Their functions are mediated in MGE-derived interneurons by the receptor Erbb4 (Yau et al., 2003; Flames et al., 2004), which can activate the MAPK or the PI3K pathway (Scaltriti and Baselga, 2006). Consistently, *in vivo* loss of Erbb4 signaling causes a reduction in the number of IN in the postnatal cortex (Flames et al., 2004).

Once INs reach the pallium, they become refractory to re-enter the subpallium (Marín et al., 2003), and colonize the cortex in a lateral-to-medial gradient (Marín et al., 2003; Tanaka et al., 2003; Cuevas et al., 2005). Individual cells move in multiple directions to disperse throughout the cerebral cortex using specific migratory routes.

Although the diverse IN types invade the cortex in largely overlapping temporal windows, this process shows a temporal gradient. The first INs that populate the cortex are the MGE-derived PV and SST neurons, as they are the first born neurons, followed by the CGE-derived VIP, CB, ChAT, and NPY expressing cells (Yozu et al., 2004; Miyoshi et al., 2010).

The different IN types migrate into the cortex *via* two main migratory routes: the intermediate zone (IZ) route is located above the germinal layers (ventricular and sub-ventricular zones) while the MZ route is close to the cortical surface (Lavdas et al., 1999; Nadarajah and Parnavelas, 2002). The choice of the migratory stream seems to depend on embryonic origins and cell identities (Kanatani et al., 2008; Antypa et al., 2011). For example, the MGE-derived interneurons PV and SST+ Martinotti cells reach the cortex *via* the MZ while non-Martinotti cells migrate *via* the IZ (Lim et al., 2018a).

The migratory process along the pallium is maintained by the action of chemokines such as CXCL12 (Li et al., 2008; López-Bendito et al., 2008), secreted from cortical cells and the meninges. This molecule activates the IN receptors CXCR4 and CXCR7, G protein-coupled receptors signaling through the inhibitory G proteins type Ga(i/o) and the MAPK-pathway, respectively and promotes migration through the migratory streams. The loss of responsiveness of INs to CXCL12 correlates with their exit from those migratory streams and the switch to the radial migration to distribute along the six layers of the cortex (Li et al., 2008; López-Bendito et al., 2008; Tanaka et al., 2010).

## Laminar Allocation

Several studies have shown that IN laminar distribution depends on the detection of cues secreted by PNs. The transition from tangential to radial migration and the allocation of IN inside the cortical plate depends on the sequential and complementary balance of at least two chemoattractive factors, CXCL12 and Neuregulin3 (Nrg3). The exit of IN from the migratory streams depends on the loss of responsiveness to CXCL12. Contrary, the final laminar distribution of INs is controlled by the PNs secreted molecule Nrg3 and its interaction with the IN receptor ErbB4 (Bartolini et al., 2017). It has been observed that in CXCR4 and CXCR7 knock-out mice INs are located in the cortical plate at early stages of brain development, sooner than observed in wild type (WT) animals (Li et al., 2008; López-Bendito et al., 2008; Wang et al., 2011). Contrary, loss of Nrg3 causes abnormal IN lamination in the adult cortex while Nrg3 overexpression promotes IN invasion of the cortical plate (Bartolini et al., 2017). Laminar allocation of CGE-derived interneurons depends also on serotonin signaling (Murphy and Lesch, 2008). Indeed, serotonin deficiency affects a set of genes involved in VIP and NPY neuron migration but leaves MGE-derived INs unaffected. This migration deficit causes persistent behavioral alterations, such as anxiety-, depressive-, and autistic-like phenotypes (Murphy and Lesch, 2008; Homberg et al., 2010).

IN laminar distribution also depends on the interaction with neighboring PNs in a cell class-specific manner (Hevner et al., 2004; Pla et al., 2006; Miyoshi and Fishell, 2011). MGE-derived INs follow an inside-out lamination pattern that correlates with birthdate, similar to PNs in the cortex (Angevine and Sidman, 1961). Interestingly, this lamination seems to be linked to that of PNs. The abnormal distribution of PNs observed in *reeler* mice (Hevner et al., 2004; Pla et al., 2006) affects the distribution of IN in a Reelin independent manner. How this applies to CGE-derived INs is unclear since they tend to occupy more superficial layers irrespectively of birthdate.

Reprograming of PNs into different subtypes by ablation of specific transcription factors (TF) causes IN lamination problems. Early born corticofugal neurons require the expression of the zinc-finger transcription factor Fezf2 (Molyneaux et al., 2005). Its absence provokes a fate switch, reprograming them into intratelencephalic neurons. Conversely, the ectopic expression of Fezf2 in intratelencephalic neurons redirects their axons to subcortical targets (Leone et al., 2008). This anormal distribution of PNs causes alterations in the laminar distribution of PV and SST interneurons (Lodato et al., 2011; Ye et al., 2015). Indeed, MGE-derived INs cluster around ectopically positioned corticofugal PNs, suggesting a major role of PNs on IN lamination and placement (Ye et al., 2015).

Similar loss-of function experiments have been recently done in intratelencephalic neurons, showing that loss of the transcription factor Satb2 transform these cells into corticofugal neurons. This causes a lamination defect on CGE-derived interneurons, as well as impairing their integration in functional circuits (Wester et al., 2019).

The end of IN migration is marked by the action of neuronal activity. While GABA has a depolarizing action on new-born INs, it causes membrane hyperpolarization once the INs reach their final destination, which decreases calcium transients and reduces interneuron motility. This hyperpolarizing effect of GABA is mediated by the expression of the potassium/chloride exchanger KCC2 (Bortone and Polleux, 2009), which allows the flux of Chloride inside the cell. The upregulation of KCC2 expression is mediated by external cues such as the neurotrophin BDNF, whose expression is restricted to PNs (Rivera et al., 2004; Rauskolb et al., 2010). BDNF also regulates the GABAergic transmission in the brain, shaping the development of neuronal circuits throughout life (Kovalchuk et al., 2004; Gubellini et al., 2005; Rauskolb et al., 2010; Sánchez-Huertas and Rico, 2011).

Altogether, these findings indicate that the laminar allocation of cortical INs depends on the correct switch from tangential to radial migration, and on the communication with surrounding PNs.

## Interneuron Integration in Brain Circuits

Inhibitory neurons are a fundamental part of brain circuitry, essential for the proper function of cortical networks. IN integration in brain circuits occurs during early postnatal days. At that time, thalamic innervation plays an important role in cortical development. Indeed, thalamic axons are critical for the maturation of cortical inhibitory circuits, which in turn are essential for the maturation of cortical networks as a whole. This early postnatal feed-forward inhibition, in which thalamic neurons excite IN which then inhibit PNs, is critical in the initial processing of sensory information (Douglas and Martin, 2004).

During the first week after birth, thalamic axons transiently innervate SST+ interneurons located in layer 5 of somatosensory cortex (Kanold and Luhmann, 2010; Marques-Smith et al., 2016) which in turn innervate PNs located in the same layer. At that time, PNs and the yet immature PV+ cells receive weaker thalamocortical inputs but strong inputs from SST+ neurons (Tuncdemir et al., 2016). Disruption of this early SST inhibitory network results in impaired maturation of thalamocortical inputs onto PV interneurons and compromises their later inhibitory capacities. This reveals a prominent role of SST+ interneurons in the maturation of deep layer cortical circuits during early postnatal development (Liguz-Lecznar et al., 2016; Marques-Smith et al., 2016; Oh et al., 2016; Tuncdemir et al., 2016; Wang et al., 2019). Consistently, silencing SST+ INs during development led to a reduction in spontaneous and sensory evoked spindle bursts (Baruchin et al., 2021). This role of SST+ interneurons in the coordination of GABAergic circuit maturation appears to be conserved in other brain regions, such as the developing hippocampus (Picardo et al., 2011; Villette et al., 2016).

PV integration and maturation also depends on paracrine mechanisms mediated by molecules such as collagen XIX (Su et al., 2020), NMDA receptors (Hanson et al., 2019), the neurotrophin BDNF and retinoic acid (RA) (Larsen et al., 2019; Lau et al., 2022). Conditional loss of collagen XIX, whose expression is enriched in multiple subtypes of SST+ interneurons (Hrvatin et al., 2018; Lim et al., 2018b) results in impaired PV perisomatic inhibition (Su et al., 2020), increasing seizure susceptibility and acquisition of schizophrenia-related behaviors (Gonzalez-Burgos et al., 2011; Lewis et al., 2011; Paz and Huguenard, 2015).

Glutamate levels are high in the neonatal cortex, leading to tonic depolarization of cortical INs but not PNs (Hanson et al., 2019). Pharmacological reduction of Glutamate signaling *via* GluN2C/D receptors lead to PV activity deficits and to cortical network hyperexcitability (Hanson et al., 2019). This suggests a role of glutamate transmission in PV maturation and assembly into cortical circuits. In line with this study, enhancement of Glutamate activity through GluN2A-subunit containing receptors improves Dravet syndrome and Alzheimer's disease in mouse models, brain disorders characterized by network hypersynchrony and cognitive impairments (Hanson et al., 2020).

The role of BDNF, synthesized and secreted by PNs (Matsumoto et al., 2008; Rauskolb et al., 2010) is another well-studied example of how feedforward communication between PNs and PV interneurons influences cortical network maturation (Huang et al., 1999; Hong et al., 2008; Sánchez-Huertas and Rico, 2011; Zheng et al., 2011; Waterhouse et al., 2012). Defects in the BDNF signaling pathway in PV interneurons has been related to disorders, such as schizophrenia (Hashimoto et al., 2005) and autism spectrum disorders (Marín, 2012). Consistently, deletion of TrkB in PV cells has recently been shown to reduce excitatory inputs onto these cells. Also, it decreases PV synapses onto surrounding cells, what decreases the influence of PV neurons in the local network (Lau et al., 2022).

Glutamatergic signaling plays a prominent role in the maturation of certain types of INs. For example, 5HTR3a INs that express reelin (RE+) and calretinin (CR+), but not VIP bipolar cells, located at the somatosensory cortex require glutamate activity for their early postnatal development (De Marco García et al., 2011). These neurons present high numbers of synapses containing NMDA receptors and receive long-range inputs from the somatosensory thalamus (Che et al., 2018). The reduction of the Glutamatergic activity on RE+ neurons by specific Knockdown of their NMDARs causes problems in the assembly of the somatosensory cortex circuit during the first postnatal week (Che et al., 2018).

Some studies have also proposed a role for Perineuronal Net proteins (PNN) in the integration of some types of INs and maturation of their synaptic properties. For example, the lack of Dystroglycan from the soma of PNs impairs the integration of CCK+ basket cells throughout the forebrain during the first postnatal week (Miller and Wright, 2021).

## Interneuron Programmed Cell Death

To ensure the assembly of functional networks in the neocortex, the brain produces an excess of neurons during development, pruning this excess once cortical networks have been properly constructed. Thus, the number of both PNs and INs is reduced during the first two postnatal weeks, reaching a steady number at the end of the second postnatal week.

The death of the PNs precedes and influences the apoptotic process of INs. PN death is regulated by glutamate signaling through NMDA receptors, whose blockage triggers apoptotic degeneration (Ikonomidou et al., 1999). This suggests a causal relationship between glutamate-dependent activity and PN apoptosis. Compared to INs, the fraction of PNs that undergo programmed cell death is minor, around 12%.

Around 30%-40% of all INs generated during development die during the first 2 weeks of postnatal life (Southwell et al., 2012; Wong et al., 2018). This refinement in INs numbers occurs after they have reached their settling positions and acquired their functional identity (Kepecs and Fishell, 2014). Such phenomenon occurs through a mechanism dependent on Bax activation (Southwell et al., 2012) and the calcium-dependent phosphatase Calcineurin (Priya et al., 2018). It has been recently reported that IN receiving strong inputs from PNs have increased possibilities to survive this period of cell death (Wong et al., 2018). The transient increment of PN activity during the period of interneuron death using DREADDs led to an increased number of MGE-derived interneurons in adult cortex. Also, the reduction of PN activity during the same period decreased the number of these INs, indicating that IN cell death is strongly linked to PN activity. This non-cell-autonomous process depends on the PN-mediated regulation of PTEN signaling in interneurons (Wong et al., 2018).

Despite that neuronal activity regulates MGE-derived IN survival, this does not seem to be the case for at least some CGE-derived INs. VIP bipolar cell survival is not regulated by neuronal activity (Priya et al., 2018). In contrast, MGE-derived interneurons seem to play a role in the apoptosis of CGE-derived neurons. The transcription factor Lhx6 is necessary for late differentiation of SST+ and PV+ interneurons and its genetic ablation causes a reduction in the number of MGE-derived interneurons (Liodis et al., 2007; Zhao et al., 2008). The loss of this IN population seems to be compensated by an increment in the number of CGE-derived interneurons (Denaxa et al., 2018).

Oligodendrocyte precursor cells (OPCs) are also indispensable regulators of interneuron populations *via* GABA signaling and the cytokine tumor necrosis factor-like WEAK inducer of apoptosis (TWEAK) (Fang et al., 2022). Ablation of GABA_B_R in OPCs is associated with an increment in the number of PV+ interneurons in all cortical layers of the adult mouse prefrontal cortex. Surprisingly, this increment in the number of PV neurons does not cause an increment of the PV inhibitory input but an impairment of interneuron activity in the medial prefrontal cortex of OPC-GABA_B_R conditional KO mice (Fang et al., 2022).

It remains still unclear if other neural cell populations control also IN programmed cell death during early postnatal days.

## Interneuron Synapse Formation

Impairments of the neuronal circuitry during development are the basis for some of the most devastating human brain disorders including schizophrenia and autism (Selemon, 2004; Fritschy, 2008; Curley and Lewis, 2012; Del Pino et al., 2013; Roux and Buzsáki, 2015). INs regulate PNs excitability and doing so they also regulate pathological hyperexcitability in the brain.

Although most glutamatergic axons preferentially connect with the dendritic spines of other projection neurons, the repertoire of GABAergic synapses is much more intricate. The different types of INs target different subcellular compartments and distribute their synapses along the entire axis of PNs (Gulyás et al., 1999; Megías et al., 2001; Hioki et al., 2013).

This process starts during development with the specification of cell fate and neuronal migration and requires multiple steps in which axonal elongation and selection of synaptic partners are both necessary. Once the neurons have reached their final location inside a cortical layer, they extend their axons to the appropriate targets within the first two postnatal weeks, followed by axonal branching and wiring occurring during the first postnatal month by still largely unknown mechanisms.

The variability on the location of synaptic contacts determines the influence on the postsynaptic cell, increasing the overall computational power of single neurons (Hauser et al., 2000; Pouille and Scanziani, 2001). While the PV+ basket and chandelier cells target the perisomatic area and the axon initial segment of the PNs to control the output of these neurons, the SST+ Martinotti INs target the distal dendrites of the PNs to control their distal inputs. On the other hand, the CGE-derived VIP+ bipolar cells mainly inhibit PV+ and SST+ interneurons, inducing disinhibition of PNs through inhibition of other INs (Tremblay et al., 2016; Lim et al., 2018a).

It is clear that the mechanisms by which the axons choose the right synaptic partner and form functional synapses (Tremblay et al., 2016; Lim et al., 2018b) requires the simultaneous orchestration of both extrinsic and intrinsic factors, and the expression of complementary molecular programs in pre- and post-synaptic neurons (Chattopadhyaya et al., 2007; Zhang et al., 2017; Favuzzi et al., 2019; Exposito-Alonso et al., 2020; Pan-Vazquez et al., 2020; Sanes and Zipursky, 2020; Steinecke et al., 2022).

### Extrinsic Factors

GABA plays a central role among the extrinsic factors that control synaptic morphogenesis and refinement (Owens and Kriegstein, 2002; Chattopadhyaya et al., 2007; Huang, 2009). It has been observed that alteration of GABA synthesis specifically in basket cells of the visual cortex causes a cell-autonomous deficit in axonal elongation and perisomatic inhibition (Chattopadhyaya et al., 2007). Conversely, overexpression of the enzyme GAD67, responsible for the production of GABA, accelerates the maturation of the perisomatic synapses (Chattopadhyaya et al., 2007). Interestingly, GABA also modulates synaptogenesis by affecting non-neuronal cells such as astrocytes and microglia cells (Nagai et al., 2019; Favuzzi et al., 2021; Gallo et al., 2022). For example, the deletion of GABA receptors specifically in microglia cells causes an increment in the perisomatic inhibition of PV+ cells onto projection neuron somas, without altering synapse specification of other neuronal populations than SST+ INs and PNs (Favuzzi et al., 2021). These data indicate that microglia cells interact with inhibitory synapses and control PV synapse formation by a GABA-mediated transcriptional program.

Glutamate-dependent neuronal activity is also required for the development of inhibitory synapse (De Marco García et al., 2011; Cserép et al., 2012; Gu et al., 2016; Hanson et al., 2019). Indeed, axon extension and dendrite arborization of putative neurogliaform cells depends on the activation of the transcriptional factor distal-less homeobox 1 (Dlx1) by glutamate signaling (De Marco García et al., 2011). Tonic activation of the NMDA glutamate receptors regulates maturation of PV+ basket cell synapses (Hanson et al., 2019), and axo-axonic synapses of ChC onto the AIS (Pan-Vazquez et al., 2020). Among the external factors that control inhibitory synapse formation and maturation of specific inhibitory synapses are the neurotrophins BDNF and NT-4 and their receptor tropomyosin-receptor kinase B (TrkB) (Rico et al., 2002; Ohba et al., 2005; Zheng et al., 2011; Xenos et al., 2018). BDNF is implicated in the maturation of inhibitory circuits (Vicario-Abejón et al., 1998; Huang et al., 1999), modulating the expression of the GABA synthetic enzyme GAD65 and the dendritic development on inhibitory (but not excitatory) neurons (Kohara et al., 2003). In mice, postnatal ablation of TrkB specifically in GABAergic interneurons of the cortex and limbic structures, causes immaturity of these type of cells, a reduction in inhibitory modulation and an increment of excitatory transmission within the prefrontal cortex (Tan et al., 2018). Furthermore, loss of the transcriptional factor Sox6 in PV+ INs affect TrkB-dependent axonal maturation and synaptic function (Munguba et al., 2021).

More recently, it has been described that the neurotransmitter Acetylcholine (ACh), which is released in the cortex *via* axonal projections from the basal forebrain, also participates in regulating axonal arborization of INs. Specifically, ACh regulates filopodia initiation in ChC cells independently of local PNs activity (Steinecke et al., 2022).

### Adhesion Molecules and Molecular Mechanisms

While most studies have focused on the role of adhesion molecules involved in axonal elongation and synaptogenesis of PNs, little is known about the molecules that control this process in INs. As in glutamatergic neurons, neurexins and neuroligins mediate GABAergic synaptogenesis (Gomez et al., 2021). The conditional deletion of neurexins in PV+ cells reduce the number of perisomatic synapses onto PNs without affecting synaptic transmission in other synapses. Surprisingly, the pan-neurexin deletion in SST+ neurons do not affect the number of synapses made by these cells but causes a decrease in synaptic strength of SST+ onto PNs (Chen et al., 2017). These results suggest that neurexins have different functions depending on the types of synapses in which they participate.

**Figure 5:**
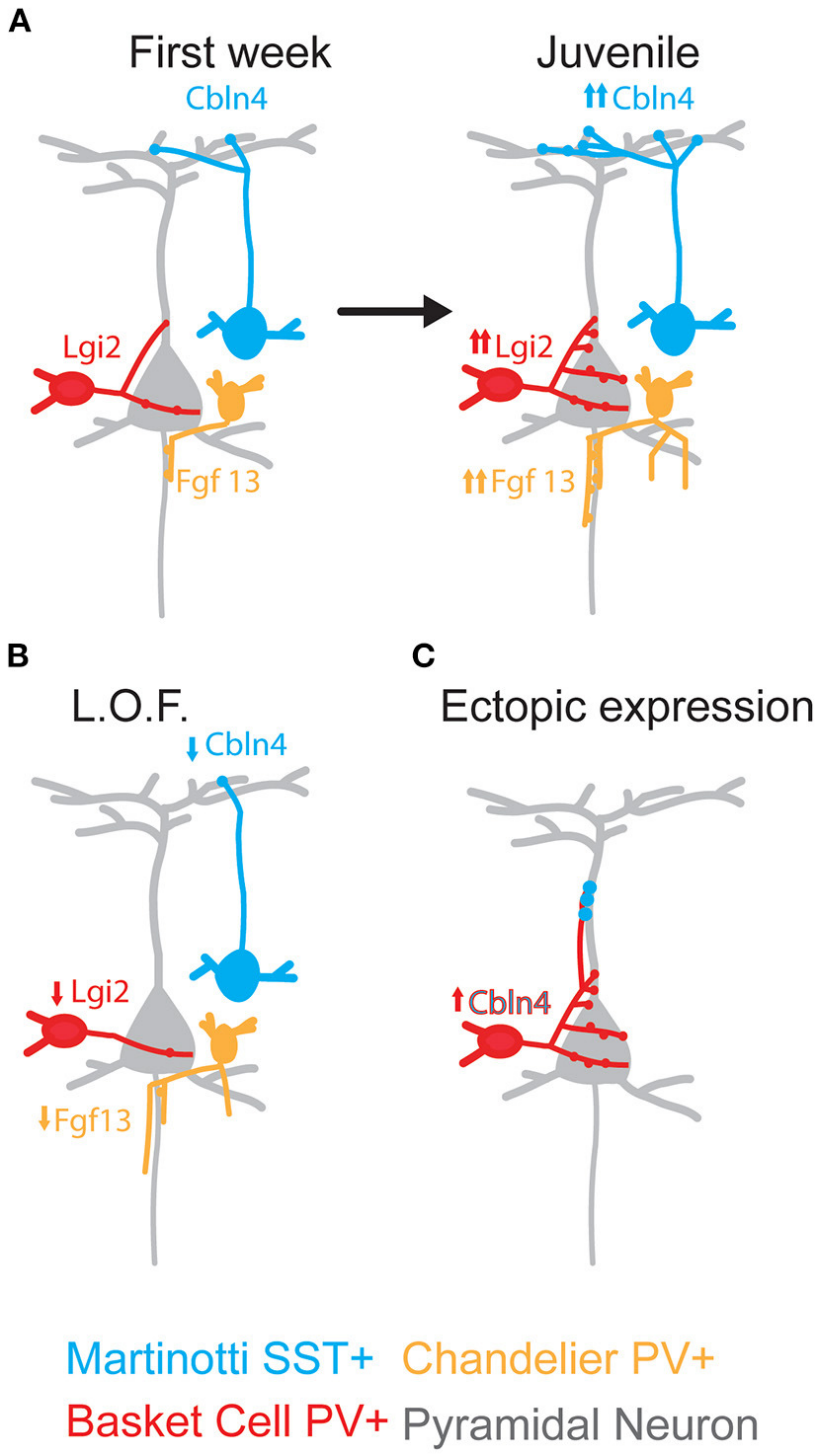
Domain-restricted synaptic molecules. **(A)** Levels for Cbln4, Lgi2, and Fgf13 increase during development following a IN-type specific molecular program that control domain-restricted synaptogenesis. **(B)** Synaptic disruption. Loss of the specific synaptogenic molecules during development causes the reduction on the number of synapses onto specific domains of Pyramidal Neurons. **(C)** Formation of domain-restricted synapses. Ectopic expression of the SST+-specific synaptic molecule Cbln4 in PV+ basket cells promotes the formation of synapses onto the dendrites of Pyramidal Neurons. Cbln4, Cerebellin4; Lgi2, Leucine-rich repeat LGI family member 2; FGF13, Fibroblast Growth Factor 13.

Recent studies using RNAseq and whole-transcriptome analyses of MGE-derived INs at the peak of synaptogenesis has shed light into the molecules involved in synaptic compartmentalization of their inputs onto PNs (Hinojosa et al., 2018; Favuzzi et al., 2019; see also Figure 5 for overview). SST+ interneurons require the participation of cerebellin-4 (Cbln4), a member of the C1q family that is a bidirectional synaptic organizer (Yuzaki, 2017). The leucine-rich repeat LGI family member 2 (Lgi2) regulates the development of basket cells perisomatic inhibitory synapses while the intracellular protein Fgf13, a microtubule stabilizer, controls axo-axonic ChC synapses onto the AIS of PNs (Favuzzi et al., 2019). Other surface proteins such as Protocadherin-18, specifically expressed in SST+ cells (Favuzzi et al., 2019), and the Neuregulins-1 and 3 (Nrg1 and Nrg3) located at the surface of PNs, also participate in the regulation of the inhibitory synapse formation in the mouse cerebral cortex (Exposito-Alonso et al., 2020). Also, the microtubule-associated kinase NEK7 regulates the wiring of PV+ interneurons (Hinojosa et al., 2018).

The high diversity of INs, the multiple factors involved in all the aspects of inhibitory synaptogenesis and the different synapses that they form onto the different compartments of PNs makes the study of specific molecules implicated in the process particularly challenging. It is clear that there is a cross talk between different INs populations and other neural cells such as PNs, astrocytes, and microglia. This makes it very difficult to understand all the process not only in normal conditions but also during pathological situations such as schizophrenia and autism-related disorders. While some adhesion molecules could be used to drive the formation of synapses onto new territories (Favuzzi et al., 2019), it is still unclear if they could have a therapeutic potential in restoring IN synaptic activity. Further studies, probably incorporating new powerful high-resolution technologies such as single cell transcriptomics should help shedding light onto these matters in the coming years.

## Discussion

After remarkable research efforts, we have achieved a quite deep understanding of IN diversity and the molecular programs controlling their migration and synaptogenesis in the mammalian cerebral cortex. The combined use of classic cell classification criteria and recent gene expression profiling seems to be bringing us closer and closer to a complete census of IN diversity in defined regions of the mammalian neocortex (Tasic et al., 2016; Gouwens et al., 2020). Likewise, the characterization of the transcriptomes has revealed the presence of specific molecular programs in different IN types, which govern the entire synaptogenic process.

In the coming years, this will probably be extended to other areas, improving our understanding of the subtle differences and particularities each local circuit seems to present. This would be of great value to understand functional consequences of IN diversity and how it adapts to circuits constructed to perform different functions.

Yet, many exciting questions about the origins of IN diversity remain open, avid of further investigation. Detailed understanding of progenitor division dynamics and associated temporal progression of genetic pathways is indispensable to understand IN development. Do MGE/CGE progenitor cells undergo complex divisions patterns, expanding ramified lineages and involving diverse progenitor cell types as observed in the LGE (Pilz et al., 2013)? If this is the case, the temporal specification of IN identities will need to be understood within that logic. For instance, nested and orthogonal molecular pathways could be expressed in different progenitor types at different points of the lineage as proposed for drosophila type II neuroblasts (Bayraktar and Doe, 2014). In any case, it seems clear that the identification of temporal sequences of gene expression, instructive of IN fates, would be inestimable to understand these processes. In addition, the competence of individual progenitor cells to generate neurons of specific types or directed to occupy distinct layers or areas remains incompletely understood. Further studies are needed to clarify to what extent the capacity to generate specific identities is segregated across progenitor cell pools or agglutinated in multipotent stem cell populations. Finally, it is worth noting that our understanding of IN fate specification remains largely superficial, mostly focused on the specification of class identities. Future studies will thus need to identify how precise IN types are specified.

Similarly, we are also starting to understand how this remarkable diversity of neurons manages to construct functional cortical networks, competent to sustain complex computations. It seems clear that different IN types deploy a different set of superficial molecules in order to establish domain-restricted synapses (Favuzzi et al., 2019). However, each individual cell type most likely requires a complex code of multiple molecules to precisely wire within the local network. For instance, while Cbln4 is required for the formation of SST+ synapses onto distal dendrites of PNs, Pchd18 seems to play an opposite role on these cells. Thus, the combined action of these two proteins is critical to balance SST cell inhibition onto PN distal dendrites. Although our understanding of the complex molecular codes that rule cortical wiring is still immature, future efforts will be of great help to dig into this matter in more detail. Such knowledge will certainly open the path for many exciting opportunities in the near future. Most importantly, perhaps, it could make us capable of driving synapses onto desired cells or territories, a skill that will likely be probed very useful both in basic research and therapeutic applications.

## Author Contributions

Both authors listed have made a substantial, direct, and intellectual contribution to the work and approved it for publication.

## Funding

This study was supported by the Spanish Ministerio de Ciencia e Innovación (PID2020-113086RB-I00 and RYC2018-025215-I) to RD. AL was funded by the Alfonso Martín Escudero Foundation.

## Conflict of Interest

The authors declare that the research was conducted in the absence of any commercial or financial relationships that could be construed as a potential conflict of interest.

## Publisher's Note

All claims expressed in this article are solely those of the authors and do not necessarily represent those of their affiliated organizations, or those of the publisher, the editors and the reviewers. Any product that may be evaluated in this article, or claim that may be made by its manufacturer, is not guaranteed or endorsed by the publisher.
